# Body Lice among People Experiencing Homelessness and Access to Hygiene Services during the COVID-19 Pandemic—Preventing Trench Fever in Denver, Colorado, 2020

**DOI:** 10.4269/ajtmh.22-0118

**Published:** 2022-07-05

**Authors:** Kristen E. Marshall, Heather E. Martinez, Tracy Woodall, Andrés Guerrero, Jessica Mechtenberg, Rachel Herlihy, Jennifer House

**Affiliations:** ^1^ Colorado Department of Public Health and Environment, Denver, Colorado;; ^2^ Centers for Disease Control and Prevention, Atlanta, Georgia;; ^3^ Council of State and Territorial Epidemiologists, Atlanta, Georgia;; ^4^ University of Colorado Denver, Denver, Colorado

## Abstract

Eight people with human body louse-borne *Bartonella quintana* infections were detected among people experiencing homelessness (PEH) in Denver during January–September 2020, prompting a public health investigation and community outreach. Public health officials conducted in-person interviews with PEH to more fully quantify body lice prevalence, transmission risk factors, access to PEH resources, and how the COVID-19 pandemic has affected resource access. Recent body lice exposure was reported by 35% of 153 interview participants. In total, 75% of participants reported reduced access to PEH services, including essential hygiene activities to prevent body lice, during Colorado’s COVID-19 stay-at-home orders. Future pandemic planning should consider hygiene resource allocation for PEH populations to prevent emerging and reemerging infections such as *B. quintana*.

## INTRODUCTION

*Bartonella quintana* is a bacterium that causes trench fever, a febrile illness most known for plaguing troops during World War I.^1^^–^^3^ Serology testing can show historical prevalence of *B. quintana* in a population; a study in France detected antibody titers in 30% of a population experiencing homelessness.^1^^,^^4^ Symptoms of *B. quintana* include headache, dizziness, leg pain, lymphadenopathy, and a relapsing fever lasting 4–8 days; severe medical complications such as endocarditis, chronic bacteremia, and bacillary angiomatosis can occur among those infected.^4^^–^^8^ Although death from *B. quintana* infection is extremely rare, a study conducted by Raoult et al. identified 12 deaths out of 101 *Bartonella* endocarditis cases (12% mortality).^9^

*Bartonella quintana*, a vector-borne infection transmitted by the human body louse (*Pediculus humanus humanus*), reemerged in Europe and North America during the 1990s. It was most common among people living in crowded conditions with a lack of access to hygiene services, particularly among people experiencing homelessness (PEH) in urban areas where conditions might facilitate disease transmission.^1^^,^^5^^–^^7^^,^^10^^–^^12^ Homeless populations have an estimated 2–30% prevalence of *B. quintana* and 19–68% prevalence of body lice.^13^^,^^14^ In 2014, Bonilla et al., found that PEH who sleep outdoors also have a statistically significant association with having body lice.^15^ Risk factors facilitating body louse infestation include poor hygiene, lack of sanitation, inadequate laundry services, sharing of clothing and bedding, crowded conditions, and high-risk behaviors (e.g., intravenous drug use).^8^ The human body louse typically lives in clothing where it can easily feed and potentially transmit *B. quintana* to its human hosts.^3^^,^^4^^,^^11^ These studies have examined body lice prevalence and risk among PEH, but have not compared these factors in sheltered versus unsheltered PEH. Although not a reportable condition in Colorado, *B. quintana* was detected among PEH in Denver during January–September 2020.

Denver’s PEH population typically sleeps overnight in outdoor encampments or indoor homeless shelters, where preventive measures for body lice transmission can be difficult to perform. The COVID-19 pandemic produced additional difficulties for PEH; cities that imposed lockdown measures to prevent spread of SARS-CoV-2 typically did not have emergency preparedness plans in place to provide additional shelter and hygiene services to PEH.^12^ Colorado issued statewide stay-at-home orders on March 26 to reduce transmission of SARS-CoV-2 during the COVID-19 pandemic, closing businesses and restricting access to essential services for PEH, including shelters, showers, laundry services, and medical providers. The Metro Denver Homelessness Initiative (MDHI) released the first State of Homelessness 2020 report showing at least 31,207 people in the Denver Metropolitan Area accessed services or housing supports for PEH during July 2019–June 2020.^16^ In the United States, 580,466 total people were experiencing homelessness during January 2020.^17^ Within this population, 9,846 people were experiencing homelessness in Colorado in January 2020. Although effects of COVID-19 on the homeless population have not yet been quantified, an economic model from Columbia University projected a 40–45% increase in homelessness in the United States by the end of 2020 despite eviction moratoriums and federal unemployment benefits.^16^^,^^18^

Because of the detection of *B. quintana* among PEH in Denver, the Colorado Department of Public Health and Environment (CDPHE) initiated an investigation. Colorado Department of Public Health and Environment sought to determine magnitude of body lice infestation among PEH in Denver, identify and compare risk factors for body lice transmission in outdoor encampments and overnight shelters, and determine how COVID-19 might have affected access to hygiene resources necessary to prevent body lice transmission. Collaborations with homeless coalitions and local public health jurisdictions sought to provide community education and outreach for body lice prevention and *B. quintana* treatment.

## MATERIALS AND METHODS

### *Bartonella quintana* detection.

During January–July 2020, local jurisdictions and hospitals where patients sought care identified and reported *B. quintana* infections to CDPHE as part of a suspected outbreak (outbreaks are considered reportable conditions in Colorado despite *B. quintana* not being reportable), with subsequent case reports occurring after CDPHE issued a Health Alert Network (HAN) notification to Colorado clinicians in July 2020 describing the illness and calling for infections to be reported. Subsequent infections were identified during August–September 2020 through retrospective hospital laboratory reviews and prospective surveillance. Confirmed *B. quintana* cases were defined by culture and positive polymerase chain reaction (PCR) laboratory testing and at least one of the following clinical symptoms compatible with *B. quintana* disease: fever (isolated or recurrent), headache, rash, bone pain (primarily in shins, neck, or back), bacillary angiomatosis (cutaneous lesions, subcutaneous masses, or bone lesions), endocarditis (evidence of valvular vegetation or embolic sequelae, such as Osler nodes and Janeway lesions), sepsis, or bacteremia.^19^^,^^20^ Probable cases were defined and classified by immunoglobulin G (IgG) serology testing, with at least one clinical symptom mentioned above, in a person experiencing homelessness.

### Data collection.

Patients with *B. quintana* admitted to hospitals before *B. quintana* detection were interviewed either in person or by telephone to collect data regarding body lice exposures and sleep location before infection. Metro Denver Homelessness Initiative provided CDPHE with data indicating when and where patients accessed day center or overnight shelter services during the previous 6 months; supplemental location data were collected from medical chart abstraction. These data were used to identify locations for a convenience sample of PEH for questionnaire administration, intervention, and education at overnight shelters, outdoor encampments, and day center facilities.

Colorado Department of Public Health and Environment created a body lice questionnaire to be administered to PEH in metropolitan areas. Participants were interviewed during September and October and included in the study sample if they were currently experiencing homelessness in Denver at one of the identified overnight shelters, outdoor encampments, or day centers. Public health officials conducted interviews during September 3–October 5 in two shelters, four outdoor encampments, and one day center offering services for PEH. Before interviews were conducted, participants were provided photos and descriptions of body lice, measures to prevent lice infestation, and *B. quintana* symptoms. Data collection included quantitative information regarding known contact with body lice during the previous 6 months, typical sleep location, access to medical care, laundry services, and shower facilities, and qualitative information regarding effects of the COVID-19 pandemic had had on access to these services. Some interview questions were not answered by participants due to participant declining, or due to interviewer decisions to abbreviate the interview due to a lack of participant cooperation. After the interview, participants were provided information on locations to access medical services should they become infested with body lice or experience *B. quintana* symptoms in the future.

Risk factors for body lice transmission were the focus for this study; certain behavioral risk factors for *B. quintana* infection, such as past or current intravenous drug use were not examined. Additionally, blood samples were not acquired from interview participants to test for *B. quintana* by PCR or serology.

### Data analysis.

Descriptive statistical analysis and univariable analysis were conducted to compare body lice prevention activities and access to services among those who only sleep in outdoor encampments (i.e., unsheltered) and those who only sleep inside in overnight shelters (i.e., sheltered). Participants who sometimes sleep in both locations were excluded due to lack of contextual data. Univariable analysis also compared those who report an exposure to body lice during the previous 6 months with those who do not. Qualitative analysis were conducted to group free text responses regarding effects of the COVID-19 pandemic had had on access to services into categories, including difficulty accessing services, business closures, less access to government and identification services, access to jobs, shelters that have moved or closed, reduced access to showers or laundry facilities, change in access to medical care, reduced transportation opportunities, and fewer food and meal options. All statistical analyses were conducted using R Studio, Version 1.2.5033^®^ (RStudio PBC, Boston, MA).

## RESULTS

Eight *B. quintana* cases among Denver’s PEH population were reported to CDPHE during January–September 2020; four cases were laboratory-confirmed by PCR and four cases met the probable case definition. All eight patients were hospitalized, five experienced endocarditis, and three experienced septicemia. One patient with *B. quintana* isolation died during hospitalization; the hospital diagnosis included valvular cardiomyopathy and infective endocarditis. Three of eight *B. quintana* patients were identified during January–February, before Colorado’s stay-at-home orders. Five of the eight patients tested positive for *B. quintana* during the months of May through September, after statewide stay-at-home orders were issued (Table 1).

**Table 1:** *Bartonella quintana* case demographics—Denver, CO, 2020

	Total
	*N* = 8
	No. (%)
Case status	
Confirmed case	4 (50)
Probable case	4 (50)
Hospitalization	
Hospitalized	8 (100)
Not hospitalized	0 (0)
Died during hospitalization	1 (13)
Endocarditis	5 (63)
Septicemia	3 (37)
Illness during stay-at-home orders	3 (37)

All categories are mutually exclusive.

### Study population.

In total, 153 interviews were completed. Interviews were conducted with 76 people currently in shelters (50%), 49 people currently in outdoor encampments (32%), and 28 people seeking PEH day center services (18%).

### Sleep location.

The study population was closely distributed when asked whether they sleep indoors at shelters (categorized as sheltered) or outdoors in encampments (categorized as unsheltered). In total, 45 (29%) participants only slept sheltered and 51 (33%) participants only slept unsheltered (Table 2). However, 46 (30%) people reported sleeping both sheltered and unsheltered and 11 (8%) stated they sleep in an alternate or undisclosed location that is not an indoor shelter or an outdoor encampment.

**Table 2:** People experiencing homelessness sleep location and human body louse (*Pediculus humanus humanus*) exposure— Denver, CO, 2020

	Total
	*N* = 153
	No. (%)
Typical sleep location	
Indoors (sheltered) only	45 (29)
Outdoors (unsheltered) only	51 (33)
Both indoors and outdoors	46 (30)
Other location	11 (8)
Body lice exposure	56 (37)
Body lice on self	23 (15)
Body lice on others	31 (20)
Body lice unspecified	2 (1)
No body lice	90 (59)
Did not disclose	7 (5)

All categories are mutually exclusive.

### Prevalence of body lice.

Body lice were frequently mistaken for head lice by study participants. No participants said they were familiar with the human body louse before the interview, but some PEH recollected exposure after being provided a description and photos of human body lice. Among 153 study participants, 54 (35%) reported exposure to body lice during the previous 6 months, whether on themselves or on others with whom they had come in contact (Table 2).

Of participants who reported having an alternative place to sleep (i.e., other), none had experienced body lice during the previous 6 months. Twenty-five percent (*N* = 14) of people who had experienced body lice on themselves or others during the previous 6 months also routinely reported sharing clothing or bedding with others.

### Access to PEH services.

Fifteen (29%) people who only sleep outside reported having access to indoor overnight shelters, compared with 39 (87%) people who only sleep indoors. However, 22 (43%) of the 15 people who only sleep unsheltered reported not wanting shelter access (Table 3). Reasons reported by people declining shelter access include mental health or social issues, such as post-traumatic stress disorder, claustrophobia, and aversion to crowds. In total, 12% of PEH participants who only sleep unsheltered perceived shelters as being dirty or having bugs and 10% reported concern for noise and crowds. No statistically significant difference was reported in access to medical care between people who only sleep unsheltered, compared with those who only sleep sheltered (98% and 87%, respectively; Table 3).

**Table 3:** Access to people experiencing homelessness services— Denver, CO, 2020

PEH services	Sheltered only*	Unsheltered only*
	*N* = 45	*N* = 51
	No. (%)	No. (%)
Access to overnight shelters		
Yes	39 (87)	15 (29)
No	2 (4)	4 (8)
Sometimes	3 (7)	1 (2)
Do not want access	1 (2)	22 (43)
Did not answer	0 (0)	9 (18)
Access to medical care		
Yes	39 (87)	50 (98)
No	4 (9)	1 (2)
Sometimes	2 (4)	0 (0)
Did not answer	0 (0)	0 (0)
Access to laundry services		
Yes	40 (89)	31 (61)
No	2 (4)	19 (37)
Sometimes	3 (7)	1 (2)
Did not answer	0 (0)	0 (0)
Share clothing or bedding		
Yes	4 (9)	11 (22)
No	34 (76)	26 (51)
Sometimes	5 (11)	5 (10)
Did not answer	2 (4)	9 (18)
Access to showers		
Yes	41 (91)	41 (80)
No	2 (4)	8 (16)
Sometimes	1 (2)	2 (4)
Did not answer	1 (2)	0 (0)

PEH = people experiencing homelessness.

* Mutually exclusive.

Nineteen (37%) people who only sleep unsheltered reported difficulty accessing laundry services, versus two (4%) of sheltered sleepers. Unsheltered participants who do not have access to laundry report they either do not launder clothes, or they launder clothes in unclean, unheated water sources such as buckets, rivers, or fountains. In total, 76% of study participants who only sleep sheltered report using in-house laundry facilities at the overnight shelters. Eleven (22%) unsheltered people also report sharing clothing or bedding with others, compared with four (9%) of sheltered people (Table 3). Among participants who only sleep unsheltered, 41 (80%) reported having access to shower facilities, compared with 41 (91%) of participants who only sleep sheltered. More sleeping sheltered respondents (69%) report showering more than once weekly than those sleeping unsheltered (31%).

### Effects of COVID-19.

During this investigation, CDPHE staff learned that several overnight shelters in Denver were reallocated to exclusive use for PEH with COVID-19 and closed to the public for several months. Other overnight shelters traditionally used by PEH were moved entirely, often to locations less accessible by public transportation in the metropolitan area. Mobile shower and laundry facilities previously available to PEH staying in encampments were discontinued during the COVID-19 shutdown, while certain other essential services (e.g., day centers and laundromats) were temporarily closed. Government services and identification document offices were also closed during Colorado’s stay-at-home orders, which presented challenges for people to acquire or replace forms of identification. Difficulties acquiring such documents restricted PEH from applying for housing, jobs, and entering overnight shelters that required identification on arrival.

**Figure 1.**
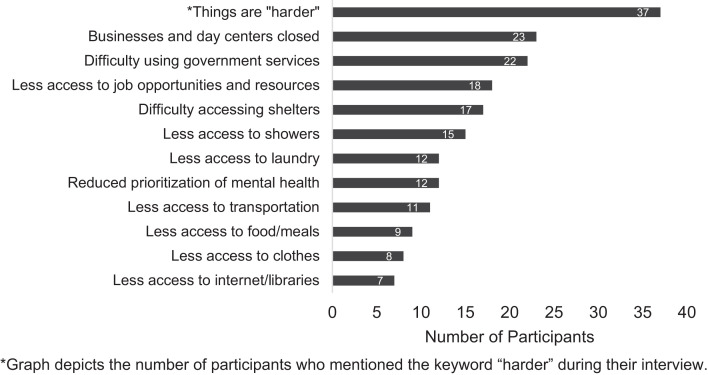
Keyword analysis describing how COVID-19 has affected access to people experiencing homelessness resources in Denver, CO.

Among 153 study participants, 115 (75%) indicated COVID-19 had affected their access to essential services and PEH resources in some way. Sleeping sheltered was not associated with participant opinions on whether COVID-19 has changed their access to resources (78% of sheltered participants and 78% of unsheltered participants, not shown). A keyword frequency analysis (Figure 1) showed the most frequent open response category among all participants who indicated COVID-19 had changed their access to resources was that things are “harder” (32%).

## DISCUSSION

*Bartonella quintana* infections had not previously been detected in Denver, but emerged prior to and during the COVID-19 pandemic in 2020. Eight PEH with *B. quintana*, a louse-borne illness often spread in areas with poor hygienic conditions, were identified in Denver during January–September 2020. A majority of PEH study participants indicated COVID-19 had affected their access to essential services, including access to laundry and shower facilities. The resultant investigation examined body lice prevalence, transmission-facilitating behaviors, and access to resources between two types of PEH sleep locations, sheltered versus unsheltered, and resulted in multiple crucial findings.

This study found that self-reported exposure to body lice is prevalent in participants who sleep in indoor shelters only, in outdoor encampments only, and in both sheltered and unsheltered locations in Denver (33%, 29%, and 41% of each category, respectively), but were not reported among participants who stated they had an alternate place to sleep, such as a motel, or with a friend or family member. In contrast, previous literature reports that body louse infestation prevalence can vary from 7% to 22% among sheltered populations or be as high as 80% among unsheltered populations experiencing poor hygienic conditions.^13^

Participants who typically sleep unsheltered report having less access to PEH services that might reduce transmission of *B. quintana* and body lice. However, fewer unsheltered participants reported recent exposure to body lice during the previous 6 months than those who typically sleep sheltered. This might be attributable to crowded conditions in shelters, compared with encampments, or to recall bias or misidentification of body lice among participants. Despite offering onsite laundry, showers, and medical care, overnight shelters still struggle with controlling human body lice transmission. A higher proportion of unsheltered participants reported high-risk behaviors for body lice transmission than those sleeping indoors at shelters. Unsheltered participants reported sharing bedding and clothing, finding new clothing, and washing clothes in unclean and unheated water sources (e.g., buckets, ponds, or rivers) more frequently than those who are sheltered with accessible onsite laundry services. The body louse prefers temperatures ranging from 84°F to 90°F, but cannot survive when the temperature exceeds 122°F.^8^^,^^21^ Hot laundering of clothing and bedding will successfully kill body lice, but unheated water sources used among PEH sleeping outdoors will not. Additionally, unsheltered participants report showering or bathing less frequently than those with onsite shower facilities at overnight shelters.

Five cases were identified during months of May–September, although body lice are typically more prevalent during colder months.^3^ While state-issued stay-at-home orders have been associated with a decrease in cases of COVID-19, they have also been shown to have some negative impacts on mental, physical, and financial health.^22^^–^^25^ The COVID-19 pandemic and Colorado’s stay-at-home orders (issued in March 2020) decreased access to PEH resources in Denver, which might have led to increased body lice and *B. quintana* transmission among this population. Approximately three quarters of participants reported experiencing negative changes to essential hygienic resources during the COVID-19 lockdown in Denver, regardless of where they typically slept.

Colorado Department of Public Health and Environment collaborated with local PEH coalitions and initiatives, local public health agencies, local hospitals and clinicians, and shelter administrative staff. Shelter staff were provided educational materials to give to PEH regarding body lice, *B. quintana* infection, prevention methods, and locations to access medical care should they obtain body lice or experience symptoms consistent with *B. quintana* infection. Educational materials and handouts regarding body lice prevention, *B. quintana* symptom recognition, medical treatment, and complications, and PEH hygienic service locations were provided to PEH encountered during site visits and interviews. Additionally, food or beverage items and socks were also offered. Healthcare providers who routinely serve Denver’s PEH population received HAN notifications providing diagnostic and treatment information for *B. quintana* and body lice infestation, and instructions to report suspect *B. quintana* patients with recent body lice exposure. Shelter and day center staff were educated on body lice prevention in their facilities and all efforts were coordinated with local coalition groups to continue outreach beyond the interview period.

This investigation was subject to several limitations. Body lice exposure was self-reported during the interview questionnaire after providing participants with a photo and description of the human body louse; subjective identification might have resulted in inaccurate body lice exposure reporting. Second, community efforts were limited to the shelters and encampments CDPHE’s investigative team was able to visit in the specified time frame. Denver has many shelters and encampments (some mobile), and the only locations visited by the investigative team were those where a confirmed or probable *B. quintana* patient had previously spent time. Categorization of participant sleep location was limited by the combined group of those who sleep both sheltered and unsheltered; this group’s interviews contained robust data, but they were ultimately unable to be properly categorized in a true comparison group due to lack of information regarding the amount of time spent sheltered versus unsheltered. The combined sheltered and unsheltered participant group is unique and provides an opportunity for future PEH research. Additionally, we were unable to quantify the number of PEH who declined to be interviewed. Some PEH were congregated into groups while interviews were conducted and occasionally an individual would decline on behalf of an entire group, or interviewers would decide not to approach certain groups. The study results may contain some bias due to the willingness of the interview participants to speak with interviewers.

Community outreach efforts helped increase awareness and understanding of body lice risk and transmission among PEH in Denver. Typically, overnight shelters offer access to laundry and shower facilities for PEH who stay there and shelter administration should prioritize body lice mitigation activities to reduce transmission among their patrons. Major metropolitan areas should include essential PEH services or allocate alternative hygienic services in preparedness and response plans for future pandemics, major events, and natural disasters to prevent vector-borne transmission of *B. quintana* and associated outbreaks.
